# Biodegradation of Dibutyl Phthalate by the New Strain *Acinetobacter baumannii* DP-2

**DOI:** 10.3390/toxics10090532

**Published:** 2022-09-09

**Authors:** Cheng Li, Chunjing Liu, Rongzhen Li, Yue Liu, Jianzhi Xie, Bowen Li

**Affiliations:** 1 College of Resources and Environmental Science, Hebei Agricultural University, Baoding 071001, China; 2 State Key Laboratory of North China Crop Improvement and Regulation, Baoding 071001, China; 3 Key Laboratory for Farmland Eco-Environment of Hebei Province, Baoding 071001, China

**Keywords:** dibutyl phthalate, biodegradation, response surface analysis, degradation pathway

## Abstract

Optimizing the culture conditions of DBP degradation by bacteria and investigating its biodegradation pathways have a great importance to develop effective PAEs pollution control strategies. In this study, we investigated the cultivation condition optimization, degradation kinetics, and degradation pathways of a newly isolated dibutyl phthalate (DBP) degradation strain, which was isolated from activated sludge and identified as *Acinetobacter baumannii* DP-2 via morphological observation, biochemical identification, and 16S rDNA sequence analysis. The degradation conditions were optimized based on the results of single-factor experiments and response surface optimization experiments. The DBP degradation rate of *Acinetobacter baumannii* DP-2 reached up to 85.86% when the inoculation amount was 17.14%, the DBP concentration was 9.81 mg·L^−1^ and the NaCl concentration was 5 g·L^−1^. The GC-MS analysis results indicated that the intermediate metabolites of *Acinetobacter baumannii* DP-2 mainly consisted of DMP, MBP, PA, and benzoic acid derivatives, which confirmed the degradation pathway from DBP to PA under aerobic pathway and then to BA under anaerobic pathway. In summary, *Acinetobacter baumannii* DP-2 shows great potential for the degradation of DBP in contaminated soils.

## 1. Introduction

Phthalate esters (PAEs) are common additives in coating materials, greasing substances, binders, pesticides, packaging, and cosmetics [1]. PAEs-based plastic films, which have been extensively used in agriculture, can cause potential pollution to soils, rivers, and oceans [2]. Some commonly used PAEs cannot undergo a stable chemical combination with plastic polymer networks [3,4] and thus can be easily released into the surrounding environment [5,6]. PAEs are considered environmental hormones with hepatotoxic and carcinogenic effects that can negatively affect reproduction, impair development, and induce genetic variations/mutations even at very low concentrations [7,8]. Many studies have provided empirical evidence that PAEs in soil can be transferred to plants and thus enter the food chain [9]. Therefore, it is of great importance to take positive and effective measure to repair environmental PAEs pollution.

Dibutyl phthalate (DBP), one of the most widely used PAEs, has been listed as a priority pollutant by the US Environmental Protection Agency, China Environmental Testing Station, and the European Union. To solve the DBP pollution problems, many studies conducted relevant research on different DBP degradation methods, including physical, chemical, and biological technologies [10]. Compared with traditional adsorption and photochemical oxidation methods, the biodegradation method is considered a better option, as it is inexpensive and does not produce secondary pollutants [11,12]. Various degrading bacteria, belonging to *Pseudomonas* sp. [11], *Gordonia* sp. [13], and *Novosphingobium* sp. [14], have been screened from different environmental surroundings, such as sediments, activated sludge, and contaminated soil [12,15]. These studies conducted extensive experiments to investigate the degradation characteristics of these strains and explore a broad spectrum of certain substrates. However, the PAEs units and concentrations in soil and water in different regions are significantly different; moreover, the optimal degradation conditions among different degrading bacteria are also different. Moreover, soil salinization is a global resource and ecological problem, especially in China. Therefore, it is of great importance to isolate new DBP-degrading bacteria with a good salt tolerance ability and further investigate the PAEs degradation pathways.

In this study, a new strain, named *Acinetobacter baumannii* DP-2 (abbreviated as DP-2), was screened and isolated from activated sludge in the aeration tank of a sewage treatment plant. This strain was identified via molecular biology techniques and morphological, physiological, and biochemical characteristic analysis. The cultivation conditions (inoculation amount, initial DBP concentration, and NaCl concentration) of DP-2 were optimized with both single-factor and surface response optimization tests. Particularly, the DBP degradation pathways of *Acinetobacter baumannii* DP-2 were investigated via GC-MS analysis.

## 2. Materials and Methods

### 2.1. Chemicals and Medium

#### 2.1.1. Chemicals

Standard Dibutyl phthalate (DBP) solution was purchased from Accustandard Co., Ltd. (Connecticut, USA). Chromatographically pure n-hexane was purchased from Thermo Fisher Scientific Inc. (Shanghai, China). All other chemical reagents in this study were of analytical grade.

#### 2.1.2. Culture Medium

The beef extract peptone medium consisted of (L^−1^) 5 g of beef extract, 10 g of peptone, and 5 g of NaCl, pH 7.2–7.4.

The mineral salt medium (MSM) consisted of (L^−1^) 5.1 g of K_2_HPO_4_, 2.5 g of KH_2_PO_4_, 2.0 g of (NH_4_)_2_SO_4_, 0.16 g of MgCl_2_, and 1.0 mL of trace elements solution.

The trace elements solution consisted of (L^−1^) 20 mg of CaCl_2_, 2.4 mg of Na_2_MoO_4_·2H_2_O, 1.8 mg of FeSO_4_·7H_2_O, and 1.5 mg of MnCl_2_·4H_2_O.

### 2.2. Isolation of DBP-Degrading Bacteria

Activated sludge samples were collected from the aeration tank of a sewage treatment plant in Zhangjiakou City, Hebei Province, China. The procedures for DBP-degrading bacteria screening were as follows: first, 10 g of activated sludge was placed into a flask containing 100 mL of MSM culture solution with a DBP concentration of 100 mg·L^−1^. Then, the activated sludge suspension was cultured at 30 °C, 150 rpm for 7 days. Subsequently, the DBP concentrations in the culture medium were gradually increased to 200, 400, 600, and 800 mg·L^−1^, and each concentration was acclimated for 1 week. Then, a small amount of acclimated bacterial suspension was dipped on a solid plate coated with inorganic salts of DBP (1000 mg·L^−1^) for isolation until a single bacterial colony was obtained. The DBP degradation ability of different pure strains was tested and compared. The strain with high and stable DBP degradation efficiency was selected as the targeted DBP degrading bacteria for further study.

### 2.3. 16S rRNA Gene Amplification, Sequencing, and Phylogenetic Analysis

The physiological and biochemical characteristics of the DBP-degrading bacteria DP-2 were analyzed following the guidelines of the “Common Bacterial Identification Manual” [16]. Strain DP-2 was identified using 16S rRNA gene sequencing. The genomic DNA of DP-2 was extracted using a bacterial genomic DNA extraction kit (31516KC4, AXYGEN USA). The primers 27F (5′-AGAGTTTGATCMTGGCTCAG-3′) and 1492R (5′-TACGGYTACCTTGTTACGACTT-3′) were used to amplify the 16S rRNA [17], and DNA sequencing was carried out by Shanghai Meiji Co., Ltd. According to the sequencing results, similarity searches and homology comparisons were performed with known gene sequences in GenBank (NCBI) to obtain related strains with high similarity. Finally, a phylogenetic tree was built using the neighbor-joining method via MEGA 6.06.

### 2.4. Optimization of Culture Conditions of DP-2

#### 2.4.1. Single-Factor Experiments

The single-factor experiments, which focused on the degradation ability of DP-2, investigated the following factors, including inoculation amount (1%, 2%, 5%, 10%, 15%, and 20%), substrate (DBP) concentration (5, 10, 20, 50, 100, and 200 mg·L^−1^), and NaCl concentration (5, 10, 20, 30, 50, and 100 g·L^−1^). Each treatment was replicated three times. The density of the DP-2 bacterial suspension was adjusted to OD_600_ =1.0 (the plate count bacterial density was approximately 3.0 × 10^9^·mL^−1^) for all the cultivation experiments. All treatments were performed in 50 mL mineral salt medium in total, and cultured at 30 °C, 150 rpm for 5 days. Liquid samples were taken every 12 h to measure DBP concentrations via GC-MS.

#### 2.4.2. Response Surface Optimization Experiments

Based on the results of the single-factor tests, a response surface optimization test was conducted to investigate the optimal degradation conditions of DP-2. The experiment was designed via Design Expert 8.06 software. The effects of inoculation amount, DBP concentration, and NaCl concentration on the degradation ability of DP-2 were investigated and optimized without additional carbon sources (Table 1).

### 2.5. Identification of the Degradation Intermediate Products of DP-2

The intermediate products identification process was conducted as follows: first, a total of 50 mL DP-2 bacterial suspension was collected after 72 hours’ cultivation and mixed with an equal volume of n-hexane in a 150 mL Erlenmeyer flask, then, the solution was ultrasonicated at room temperature for 30 min. A separatory funnel was used to remove the lower organic phase. Approximately 4 g of anhydrous sodium sulfate was added, and the solution was allowed to dry until the volume was 0.5 mL. Finally, the solution was diluted to a total volume of 1 mL with dichloromethane and transferred to a brown sample bottle for measurement.

Gas chromatography mass spectrometry (GC-MS, 7890B-7000C, Agilent, Palo Alto, USA) was performed on an Agilent HP-5MS UI column (30 m × 0.25 mm × 0.25 μm). The inlet temperature was set to 280 °C. The initial column temperature was set to 80 °C and held for 1 min. Afterward, the temperature was increased to 280 °C at a rate of 20 °C·min^–1^ and kept for 4 min. Helium gas was added at a flow rate of 1 mL·min^−1^. GC-MS was performed via splitless injection at an injection volume of 1 μL. An ion source mode at 300 °C was used. The quadrupole temperature was set at 150 °C. The MSD transmission line temperature was set at 300 °C. The electron energy was 70 eV. The scanning range was 50–550 *m*/*z*.

### 2.6. Calculations

The DBP degradation data of DP-2 are fitted via the first-order kinetic model according to the following equation:

$$C_{t}=C_{0}e^{-kt}$$

$$t_{1/2}=\frac{\ln2}{k}$$

where *C_t_* is the total DBP concentration in the system at *t* (d) (mg·L^−1^), *C_0_* is the initial total DBP concentration (mg·L^−1^), *k* is the first-order kinetic parameter of microbial degradation (d^−1^), and *t*_1/2_ is the half-life (d).

## 3. Results

### 3.1. Identification and Characterization of DP-2

In this study, a total of seven strains which can utilize DBP as the sole carbon and energy source were isolated from the activated sludge samples in a waste water treatment plant in Hebei, China. Strain DP-2, which was stored in the China General Microbiological Culture Collection Center (number as CGMCC No. 22236), showed the highest DBP degradation rates and was selected as the test microorganism for further cultivation optimization and DBP biodegradation pathway study.

**Figure 1:**
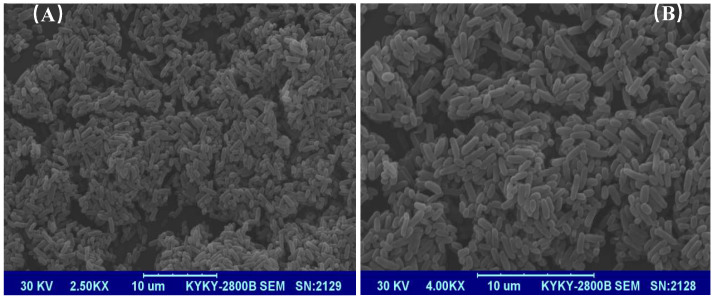
SEM images of strain *Acinetobacter baumannii* DP-2 at (**A**) 2.5KX and (**B**) 4.0KX.

**Figure 2:**
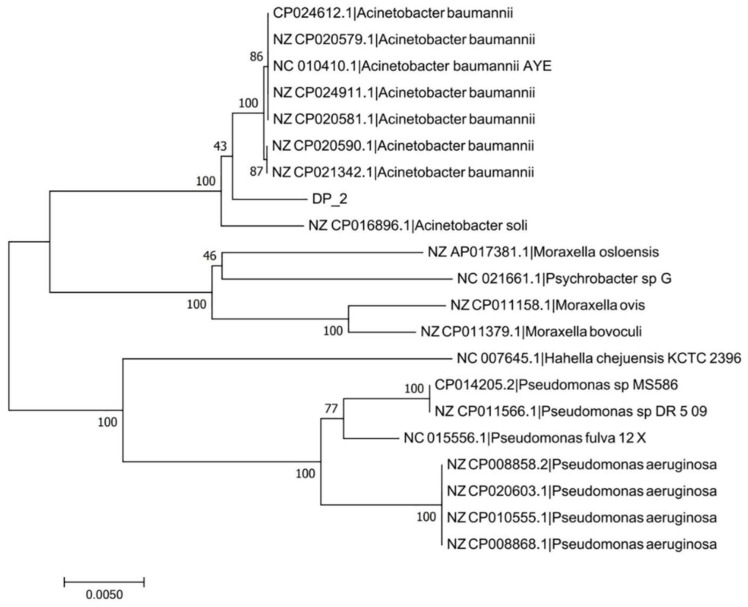
Phylogenetic tree of *Acinetobacter baumannii* DP-2 based on 16S rDNA sequences.

Strain DP-2 was identified according to the morphological observation, biochemical identification, and 16S rDNA sequence analysis. Its physiological and biochemical characteristics are summarized in Table 2. It is a Gram-negative bacterium with a short rod shape (Figure 1). The 16S rDNA region sequences of DP-2 were aligned to the NCBI database. The phylogenetic tree constructed via MEGA 6.06 software shows that DP-2 has the closest genetic relationship with *Acinetobacter baumannii* (Figure 2).

### 3.2. Effects of Culture Conditions on the DBP Degradation Rates of DP-2

#### 3.2.1. Inoculation Amount

**Figure 3:**
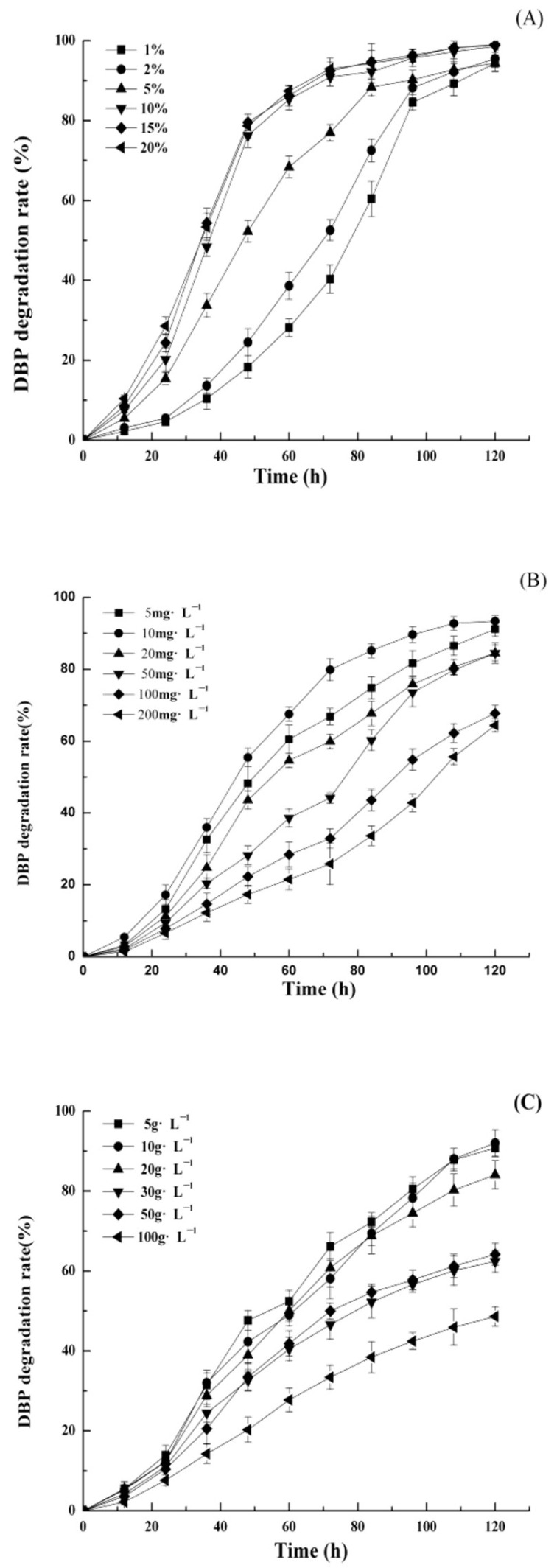
The DBP biodegradation rate of *Acinetobacter baumannii* DP-2 with different (**A**) inoculum amounts, (**B**) substrate concentrations, and (**C**) NaCl concentration.

The effects of inoculation amount on the DBP degradation rates of DP-2 were investigated at an initial DBP concentration of 10 mg·L^−1^. The results indicate that DBP degradation rates greater than 90% were achieved in all treatments after 5 days’ cultivation when the inoculation amount ranged from 1% to 20% (Figure 3A). It showed no significant differences when the inoculation amounts were 10%, 15%, and 20%. However, DP-2 showed relatively lower DBP degradation rates when the inoculation amount was lower than 5%. Thus, to achieve higher DBP degradation rates, the inoculation amount of DP-2 should be kept at least 10% during the cultivation process.

#### 3.2.2. DBP Concentration

The experiment results indicated that the initial DBP concentration, which ranged from 5–200 mg·L^−1^, can affect the DBP degradation rates of DP-2 to a certain extent. Among all the treatments, the maximum DBP degradation rate was 98.89% when the initial DBP concentration was 10 mg·L^−1^. DP-2 exhibited a greatly higher DBP degradation rate that reached more than 90% when the initial DBP concentration was in the range of 5–50 mg·L^−1^ (Figure 3B). However, the DBP degradation rate of DP-2 decreased to 60% when the initial DBP concentration was 100 mg·L^−1^, and its DBP degradation rate was even lower at a DBP concentration of 200 mg·L^−1^.

#### 3.2.3. NaCl Concentration

Salinity plays an important role in regulating osmotic pressure in microorganisms. The impacts of NaCl concentration on the DBP degradation rate of DP-2 were investigated when the NaCl concentration was in the range of 5–100 g·L^−1^. They showed the NaCl concentration had a significant effect on the DBP degradation rate of DP-2 (Figure 3C). The DBP degradation rate of DP-2 decreased as the NaCl concentration increased. The DBP degradation rate of DP-2 attained the maximum value of 97.83% when the NaCl concentration was 5 g·L^−1^, whereas the DBP degradation rate was only 50% when the NaCl concentration was 100 g·L^−1^. Overall, the NaCl concentration should be kept in the range of 5–20 g·L^−1^ to ensure a higher DBP degradation rate of DP-2.

### 3.3. Optimization of the Degradation Performance of DP-2 via the Response Surface Method

The parameters, including inoculation amount, DBP concentration, and NaCl concentration, were selected as the variables to optimize the degradation performance of DP-2 via a Box–Behnken response surface experiment. The results of the response surface test are summarized in Table 3. The corresponding quadratic equation model is as follows:

$$\eta_{DBP}=73.97-8.18A+1.12B-5.28C+0.11AB+1.42AC-0.13BC+3.22A^{2}-1.23B^{2}-9.38C^{2}$$

where A is the NaCl concentration (g·L^−1^), B is the inoculation amount (%), and C is the initial DBP concentration (mg·L^−1^).

The results show that, in the first term of the equation, A was a very significant factor and C was a significant factor (Table 4). The order of factors affecting the degradation ability of DP-2 was A > C > B. In the second term of the equation, C was a very significant factor, and the interaction terms among the parameters were not significant. The multiple-regression relationship was significant in that the correlation coefficient (R^2^) was 0.9107 and the misfit term was 0.1013, indicating that the misfit was not significant. These results show that the equations are well fitted, and the model can reflect the true relationship between each factor and the response value.

**Figure 4:**
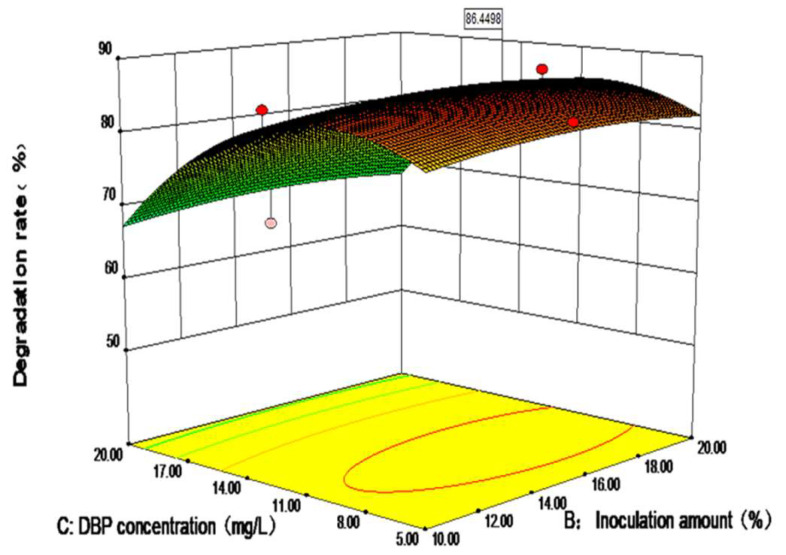

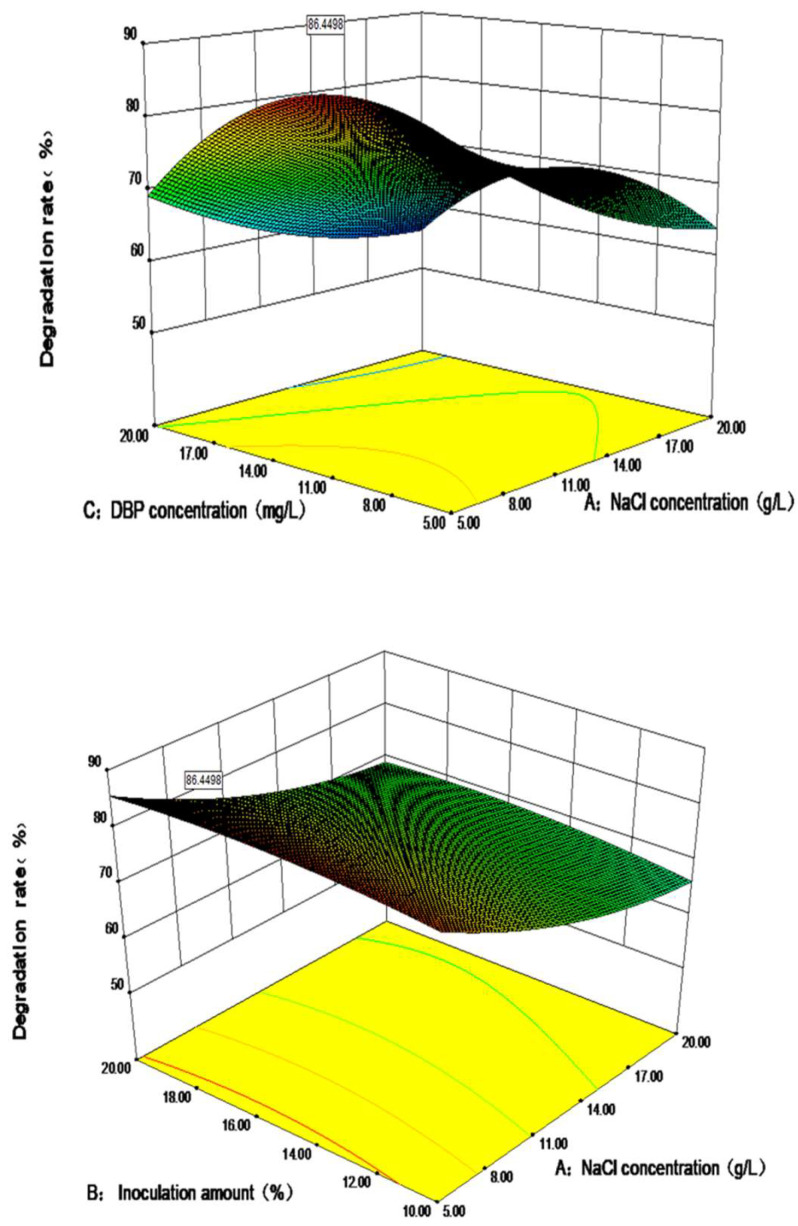
Response surface graph on the DBP degradation rate of *Acinetobacter baumannii* DP-2.

The experimental data were input into Design Expert software to create 3D and contour maps (Figure 4). The interaction between inoculation amount and DBP concentration affected the growth of DP-2 when the NaCl concentration was 5 g·L^−1^. DP-2 exhibited higher DBP degradation rates when the inoculum amount was within the range of 10–20% and the DBP concentration was 8–14 mg·L^−1^. When the DBP concentration was fixed, the inoculum amount had minimal effects on the DBP degradation rate. However, when the inoculum amount was constant, the DBP concentration exerted a substantial effect on the DBP degradation rate. In general, the influence of these factors on the DBP degradation rate initially increased and then decreased. The contour map and results of the significance analysis revealed that the interaction between inoculum amount and substrate concentration was not obvious.

Based on the above analysis, the best degradation conditions for DP-2 were as follows: Nacl concentration of 5 g·L^−1^, inoculum amount of 17.14%, and substrate concentration of 9.81 mg·L^−1^. The theoretical maximum value of the DBP degradation rate predicted by the model was 86.45%. The reliability of the model result was verified by testing the optimal degradation conditions three times. The average DBP degradation rate measured in three parallel experiments was 85.86%, which was close to the model predicted value, indicating that the model can better simulate and optimize the degradation conditions of strain DP-2. Moreover, the result verified the feasibility of optimizing the degradation conditions of strain DP-2 via the response surface method.

### 3.4. The DBP Biodegradation Kinetics of Acinetobacter baumannii DP-2

The biodegradation kinetics of *Acinetobacter baumannii* DP-2 were investigated in MSM using DBP as the substrate under the optimized culture conditions. When the initial DBP concentration was 5, 10, 20, 50, and 100 mg·L^−1^, the corresponding degradation rates after 120 h of cultivation were 98.89%, 97.82%, 92.63%, 90.46%, and 88.28%, respectively (Table 5). The results fit the first-order kinetic model well (R^2^ > 0.9631), and the half-life ranges varied from 15.91 h to 60.26 h.

### 3.5. The DBP Biodegradation Pathway of Acinetobacter baumannii DP-2

**Figure 5:**
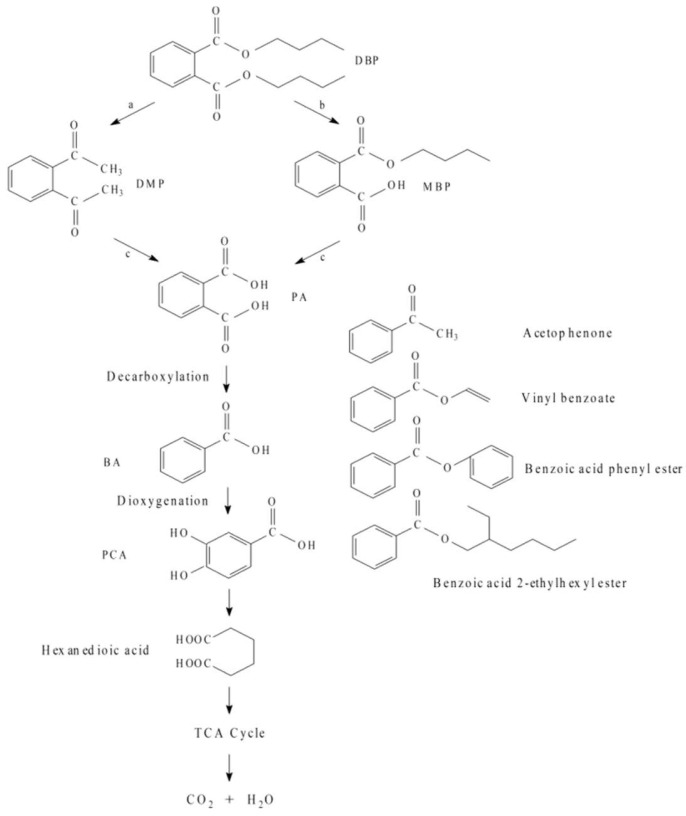
The proposed DBP biodegradation pathways of *Acinetobacter baumannii* DP-2 on the basis of metabolite analysis by GC-MS.

To understand the DBP biodegradation pathway of *Acinetobacter baumannii* DP-2, the biodegradation intermediates of DP-2 after 72 h of cultivation were extracted and identified by GC-QQQMS. Using accurate mass measurements, the chemical compositions of all possible metabolites in the medium were identified based on the NIST11 database (Figure S1). Metabolites including DBP (*m*/*z* = 149), DMP (*m*/*z* = 163), MBP (*m*/*z* = 149), and PA (*m*/*z* = 104) were detected. The main intermediate products, including PA, DMP, and MBP, appeared at retention times of 14.184, 16.029, and 21.994 min, respectively. This implied the first biodegradation step of DBP by *Acinetobacter baumannii* DP-2, which involved two possible reaction pathways (Figure 5). On the one hand, DBP could be degraded through pathway a (DBP→DEP→DMP→PA), in which DBP was first degraded to form DMP through β-oxidation and demethylation and then further hydrolyzed to produce PA; on the other hand, DP-2 was capable of degrading DBP through aerobic action via pathway b (DBP→MBP→PA). In this process, DBP was biodegraded to MBP through hydrolyzation and then further hydrolyzed to produce PA.

In addition, four new peaks were detected at retention times of 10.907, 13.619, 19.063, and 19.75 min, which corresponded to acetophenone, vinyl benzoate, benzoic acid phenyl ester, and benzoic acid 2-ethylhexyl ester, respectively. These substances were considered benzoic acid derivatives. This implied that the accumulated metabolites may be converted to benzoic acid by decarboxylation. Moreover, non-accumulated intermediates were found within 24–120 h, indicating that the intermediate metabolites MBP, DMP, and PA were degraded effectively by *Acinetobacter baumannii* DP-2.

## 4. Discussion

### 4.1. The DBP Biodegradation Ability of Acinetobacter baumannii DP-2

In this study, strain *Acinetobacter baumannii* DP-2 was isolated from the activated sludge samples in a sewage treatment plant; it can use DBP as its sole carbon source and degrade it completely. The strain *Acinetobacter* sp. M673, which was also isolated from the sewage outlet of a sewage treatment plant, can degrade various types of PAEs (DBP, DPP, and DHP), but it cannot fully utilize the intermediate metabolites [18]. *Acinetobacter* sp. strain SN13 was also isolated from activated sludge collected from a regional sewage treatment plant in the Macao Special Administrative Region of China. It exhibits high degradation efficiency at an initial DEHP concentration of 400 mg·L^−1^; however, its degradation rates can be inhibited by high DEHP concentrations [19]. *Acinetobacter* sp. strain LMB-5, which was isolated from vegetable greenhouse soil, can efficiently degrade DMP, DEP, and DBP by 98.87%, 94.94%, and 72.15%, respectively. However, its DEHP degradation rate was slow because of steric hindrance [20]. A new endophytic strain, *Bacillus megaterium* strain YJB3, was successfully isolated from canola root tissues [3]. Its DBP degradation rate reached up to 82.5% within 5 days. The strain *Pseudomonas* sp. V21b was isolated from a municipal solid waste leachate sample. Its DBP degradation rate was only 57% at an initial DBP concentration of 1994 mg·L^−1^. Different strains exhibit different PAE degradation abilities and degradation efficiencies, which are affected by certain proteins encoded by their own genes and various environmental factors. Most degrading bacteria have highly efficient degradation ability and environmental adaptability. Moreover, they can tolerate high PAEs concentrations. However, the concentration of pollutants in the actual environment is often low (mostly below 10 mg·L^−1^) [21], resulting in a failure to induce the expression of functional enzymes of the degrading strains. Furthermore, low concentrations of pollutants further lead to very low bioavailability to maintain the normal growth of bacterial cells. Therefore, it is difficult for bacteria to degrade PAEs in real polluted surroundings [21,22]. In this study, *Acinetobacter baumannii* DP-2 exhibits excellent DBP-degrading performance even when the DBP concentration was lower to 10 mg·L^−1^. This provides a theoretical reference for the further study on screening of functional genes during the PAEs-degrading process.

### 4.2. Biodegradation Pathways of Acinetobacter baumannii DP-2

Identifying the biodegradation pathways of PAEs-degrading microorganisms is crucial for the application of microbial remediation technology. The main intermediate metabolite rate-limiting step can be found by analysis of the cultivation medium by GC-MS. In this study, *Acinetobacter baumannii* DP-2 can convert DBP (*m*/*z* = 149) to DMP (*m*/*z* = 163) via β-oxidation and transesterification and then through deesterification hydrolysis to produce PA (*m*/*z* = 104). In addition, *Acinetobacter baumannii* DP-2 can hydrolyze DBP to MBP (*m*/*z* = 149) and then further to PA. The above degradation pathways of DP-2 resulting in PA formation are all aerobic biodegradation pathways [23]. However, the GC-MS results indicated that *Acinetobacter baumannii* DP-2 converted PA to benzoic acid through an anaerobic pathway. Four benzoic acid derivatives were detected in the cultivation medium in this study. It has been reported that the intermediate metabolite PA can be transformed to BA by facultative anaerobes under aerobic conditions [24]. It also reported that PA was biodegraded to BA by *Bacillus mojavensis* B1811, which is a facultative anaerobe [25]. The above findings were in accordance with the results in this study. This proved that *Acinetobacter baumannii* DP-2 can grow under both aerobic and anaerobic conditions, indicating its facultative growth characteristics. In the early stage of cultivation, *Acinetobacter baumannii* DP-2 converts DBP in the aerobic pathway when oxygen is sufficient. With the degradation process ongoing, the oxygen concentration gradually decreases, leading to an anaerobic environment within the cultivation system; hopefully, *Acinetobacter baumannii* DP-2 degrades the intermediate metabolites under anaerobic pathways. Protocatechuate (PCA), which is considered an important metabolite for PAE biodegradation, unfortunately has not been detected in this study. The reason might be the fast biodegradation rates of DP-2 once it forms. Generally, PCA can be degraded by ring-cleaving enzymes to produce adipic acid, which has been detected. Then, the degradation product of DBP enters the TCA cycle after PCA is cracked [23].

For the biodegradation of PAEs, the conversion of PAEs into PA has been reported as the key step for bacterial degradation [24,26,27]. Most PAEs and PAEs-degrading intermediates may lead to mutation, developmental toxicity, and multiplication toxicity [28]. Moreover, some of these pollutants are regarded as persistent pollutants. Therefore, a safe biodegradation technology should not induce high concentrations of such intermediates. As reported, some strains can only degrade PAEs into PA, since these strains cannot use PA as a carbon source for cell growth [29]. In this study, the peak areas of MBP, DMP, and PA were considerably smaller than those of DBP, and no accumulated intermediates were found within 24–120 h, indicating that MBP, DMP, and PA can be used as carbon sources by strain DP-2 to degrade DBP effectively. Therefore, *Acinetobacter baumannii* DP-2 can carry out the complete biodegradation of DBP itself, which means that DP-2 can be considered a promising strain for engineering applications.

## 5. Conclusions

The DBP-degrading bacterium *Acinetobacter baumannii* DP-2, which was isolated from activated sludge, can utilize DBP as its sole carbon source and effectively degrade DBP. The DBP degradation conditions were optimized via single-factor experiments and response surface optimization experiments with a DBP degradation rate up to 90% after 5 days of cultivation. The degradation intermediates DMP, MBP, and PA were detected via GC-MS, thus verifying the pathway by which *Acinetobacter baumannii* DP-2 degrades DBP. Moreover, the present study identified derivatives of benzoic acid and related intermediate products during the DBP degradation process, which shows that *Acinetobacter baumannii* DP-2 is a facultative bacterium. Thus, *Acinetobacter baumannii* DP-2 exhibits great application potential for degrading PAEs in the environment.

## Figures and Tables

**Table 1:** Box-Behnken design factor level.

Factor	Code	Level		
		−1	0	1
NaCl concentration (g·L^−1^)	A	5	10	20
Inoculum (%)	B	10	15	20
Initial DBP concentration (mg·L^−1^)	C	5	10	20

**Table 2:** Physiological and biochemical characteristics of *Acinetobacter baumannii* DP-2.

Physiological and Biochemical Indexes	Test Results	Physiological and Biochemical Indexes	Test Results
Glucose fermentation (acid production)	+	Hydrogen peroxide	+
Glucose fermentation (gas production)	−		
Fructose fermentation (acid production)	+	Nitrate reduction	−
Oxidase	−	Urease	−
Sucrose fermentation	+	Methyl red experiment	+
Mannose fermentation	+	Acetyl methanol test	−
Mannitol fermentation	+	Xylose fermentation	−
Hydrogen sulfide production	−	Gelatin liquefaction	−
Lactose fermentation	−	Tween 80	−
Arabinose fermentation	−	Starch hydrolysis	+
Gossypose fermentation	−	Indole experiment	−
Inositol	−	Citrate utilization	+

“+”: Positive reaction; “−”: Negative reaction.

**Table 3:** The degradation optimization of DP-2 based on Box-Behnken experiments.

No.	A (Inoculum Amount)	B (Initial DBP Concentration)	C (NaCl Concentration)	Degradation Rate (%)
1	1	1	0	66.78
2	0	1	1	62.23
3	−1	0	1	64.05
4	0	−1	1	60.11
5	−1	0	−1	83.64
6	1	−1	0	64.48
7	0	0	0	74.43
8	1	0	1	54.83
9	−1	1	0	86.23
10	−1	−1	0	85.36
11	0	1	−1	66.87
12	1	0	−1	68.73
13	0	0	0	73.66
14	0	0	0	75.23
15	0	0	0	72.97
16	0	0	0	73.56
17	0	−1	−1	64.22

**Table 4:** ANOVA analysis for the response surface quadratic model of DBP degradation.

Source	Sum of Squares	d_f_	Mean Square Error	F-Value	*p*-Value	Significance
Model	1189.37	9	132.15	7.93	0.0062	*
A-salinity	535.63	1	535.63	32.16	0.0008	**
B-inoculum	9.99	1	9.99	0.60	0.4640	
C-substrate concentration	223.03	1	223.03	13.39	0.0081	*
AB	0.046	1	0.046	2.775 × 10^−3^	0.9595	
AC	8.09	1	8.09	0.49	0.5082	
BC	0.070	1	0.070	4.216 × 10^−3^	0.9500	
A^2^	43.76	1	43.76	2.63	0.1491	
B^2^	6.38	1	6.38	0.38	0.5555	
C^2^	370.56	1	370.56	22.25	0.0022	**
Residual error	116.60	7	16.66			
Lack of fit items	113.53	3	37.84	49.42	0.1013	
Pure error	3.06	4	0.77			
Total error	1305.96	16				

Note: * means statistically significant at the 95% confidence level (*p* < 0.05); ** means statistically significant at the 99.95% confidence level (*p* < 0.005).

**Table 5:** Degradation kinetics equations of *Acinetobacter baumannii* DP-2 at different initial DBP concentrations.

Initial Concentration (mg·L^−1^)	Kinetic Equation	Kinetic Parameter (*K*/h^−1^)	t_1/2_/h	R^2^
5	ln*C* = −0.0193*t* + 1.8746	0.0193	15.91	0.9927
10	ln*C* = −0.0277*t* + 2.7451	0.0277	25.02	0.9869
20	ln*C* = −0.0191*t* + 3.3530	0.0191	36.28	0.9856
50	ln*C* = −0.0187*t* + 4.3777	0.0187	37.06	0.9631
100	ln*C* = −0.0115*t* + 4.8737	0.0115	60.26	0.9641

## Data Availability

The data that support the findings of this study are available from the corresponding author upon reasonable request.

## Supplementary Materials

The following supporting information can be downloaded at: https://www.mdpi.com/article/10.3390/toxics10090532/s1, Figure S1: The GC-MS analysis of the metabolites produced from DBP degradation by *Acinetobacter baumannii* DP-2.
