# Supplementary material for: Biodegradation of Dibutyl Phthalate by the New Strain Acinetobacter baumannii DP-2
Source: Toxics. 2022 Sep 9;10(9):532. doi: 10.3390/toxics10090532 (PMC9505308; doi:10.3390/toxics10090532)
Supplement: Supplementary file 1 [file toxics-10-00532-s001.zip › toxics-1893717-supplementary.pdf]

Supplementary Materials: Biodegradation of Dibutyl Phthalate by the New Strain Acinetobacter baumannii DP-2

Cheng Li, Chunjing Liu, Rongzhen Li, Yue Liu, Jianzhi Xie and Bowen Li

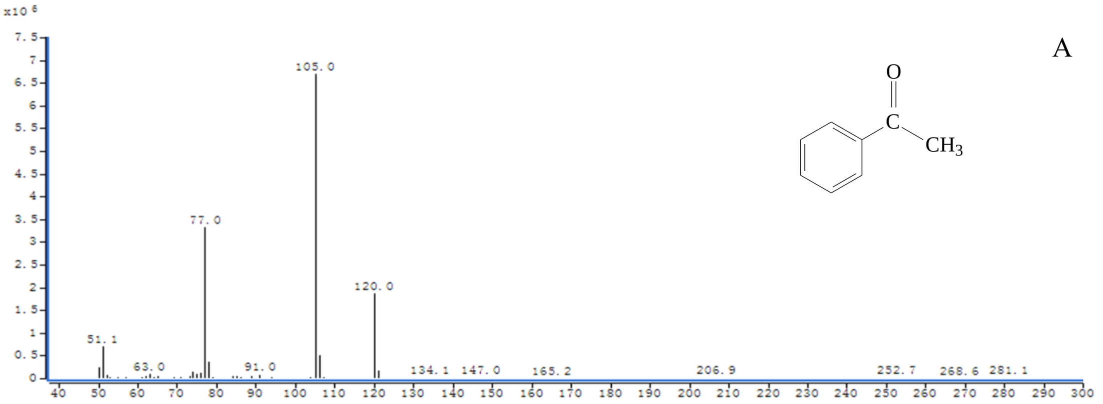

SCAN:10.907min

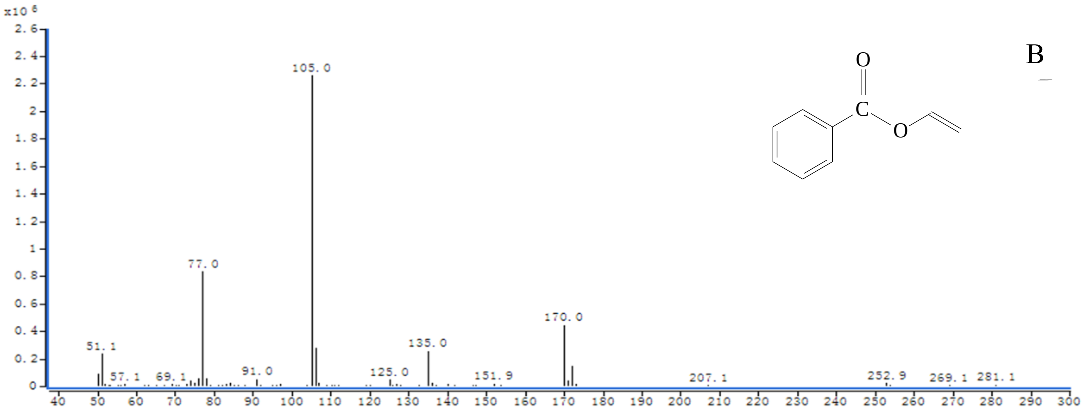

SCAN:13.619min

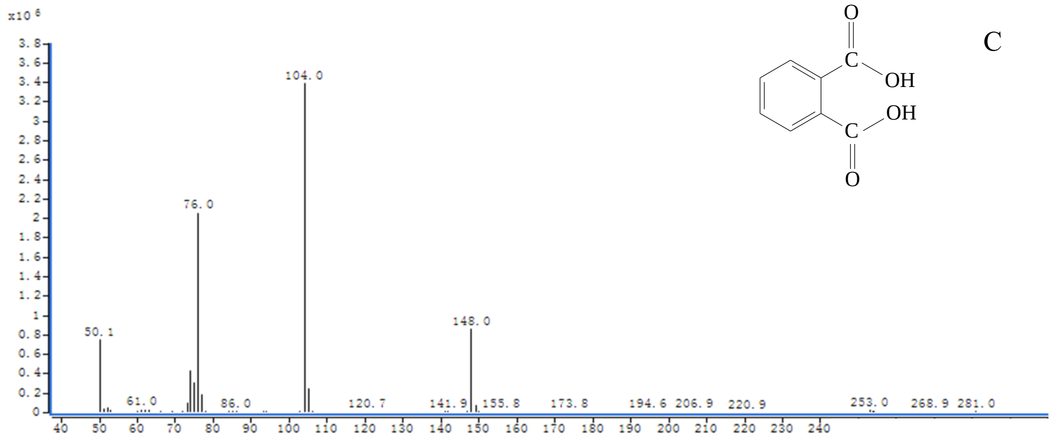

SCAN:14.184min

D

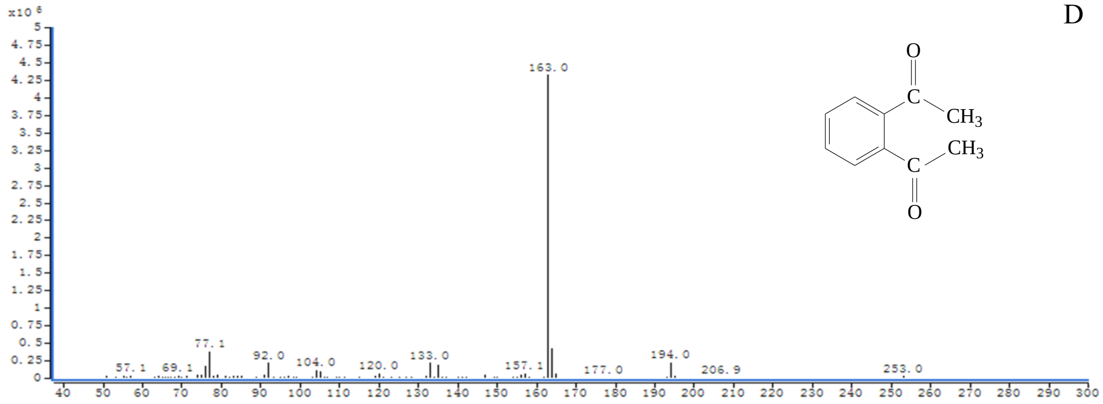

SCAN:16.029min

E

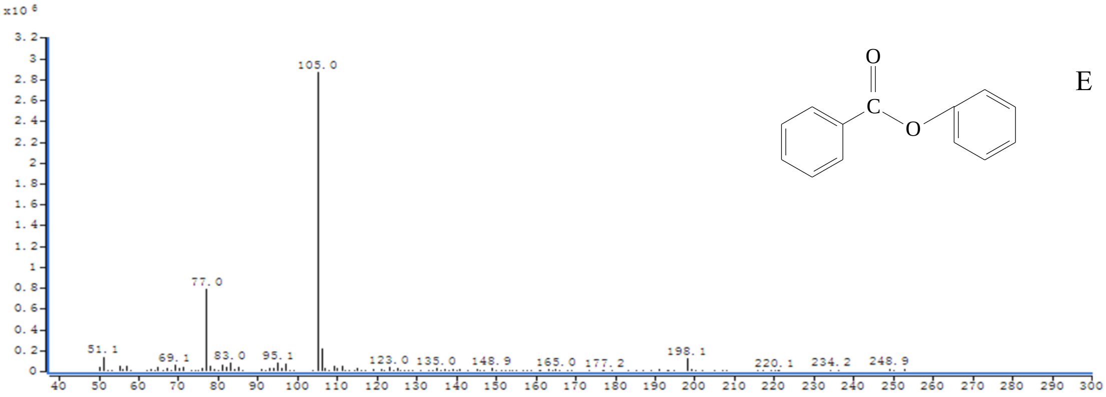

SCAN:19.063min

F

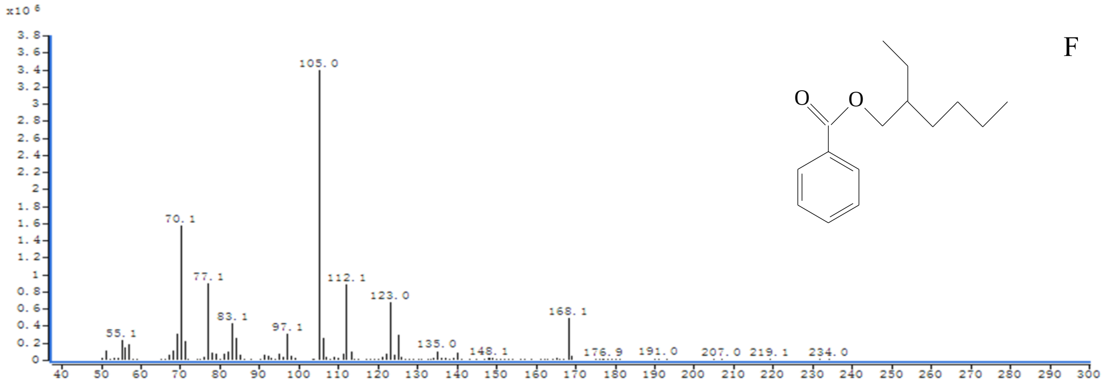

SCAN:19.751min

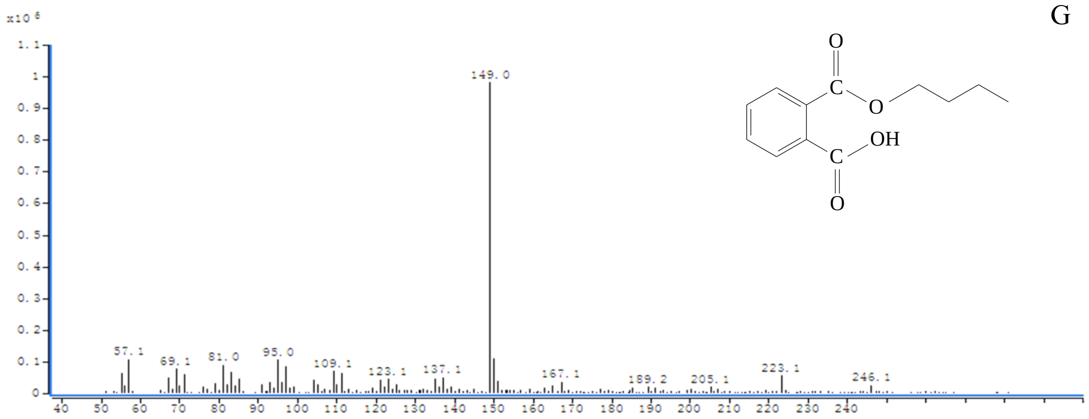

SCAN:21.994min

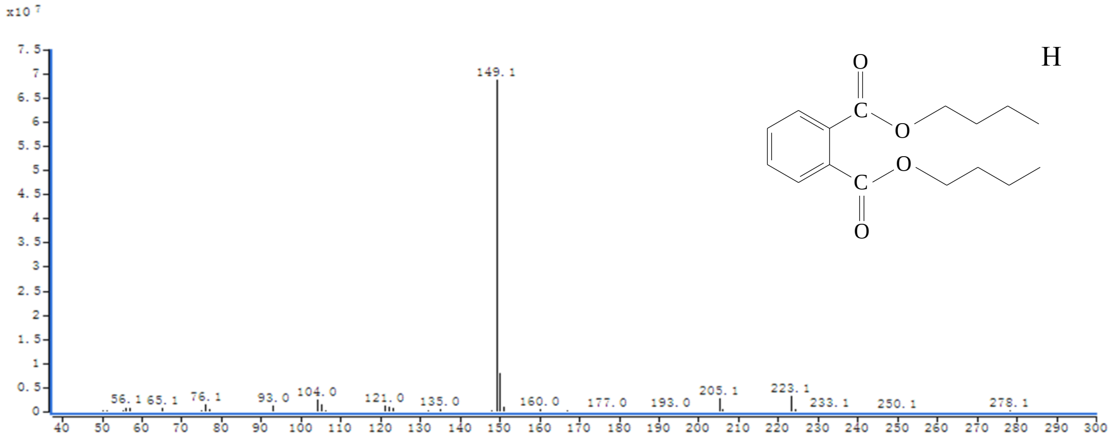

SCAN:23.356min

Figure S1. The GC-MS analysis of the metabolites produced from DBP degradation by Acinetobacter baumannii DP-2. Characteristic ions of compounds A–H in GC-MS. The retention times of the compounds were 10.907, 13.619, 14.184, 16.029, 19.063, 19.751, 21.994 and 23.356 min, respectively, which were identified as **A**: Acetophenone; **B**: Vinyl benzoate; **C**: 1,2-Benzenedicarboxylie acid (PA); **D**: Dimethyl phthalate (DMP); **E**: Benzoic acid, phenyl ester; **F**: Benzoic acid, 2-ethylhexyl ester; **G**: mono-n-butyl phthalate (MBP); H: Dibutyl phthalate (DBP).
